# The influence of sintering of osteoporotic vertebral fractures on the sagittal lumbar profile and degenerative changes

**DOI:** 10.1186/s13018-025-05454-3

**Published:** 2025-01-09

**Authors:** Christoph Beyersdorf, Max Prost, Melanie Elisabeth Röckner, Uwe Martin Maus, Cornelius Jacobs, Max Joseph Scheyerer

**Affiliations:** 1 Department of Orthopaedic and Trauma Surgery, Medical Faculty, University Hospital Düsseldorf, Heinrich-Heine-University, Düsseldorf, Germany; 2 Department of Orthopaedic and Trauma Surgery, St. Remigius Hospital Leverkusen, Leverkusen, Germany

**Keywords:** Osteoporosis, Vertebral body fracture, Degenerative spine disease, Spinal stenosis, Spinal surgery

## Abstract

**Background:**

Osteoporosis, a skeletal disorder affecting nearly 20% of the global population, poses a significant health concern, with osteoporotic vertebral body fractures (VBF) representing a common clinical manifestation. The impact of osteoporotic sintering fractures in the thoracolumbar spine on the sagittal lumbar profile is incompletely understood and may lead to the onset of clinical symptoms in previously asymptomatic patients.

**Methods:**

This retrospective single-center study analyzed data from patients presenting with osteoporotic spine fractures between 2017 and 2022. Patient selection involved stringent inclusion and exclusion criteria, focusing on radiologically documented osteoporotic sintering fractures in the thoracolumbar junction (TH11-L2). Clinical parameters were recorded and analyzed, alongside lateral-view radiographic assessments utilizing the IDS 7-PACS^®^-System (Sectra, Linköping, Sweden). Measurements included total lumbar lordosis, lordosis caudal to the fracture, kyphosis of the fractured vertebra, and sacral slope. Statistical analysis was conducted using SPSS 27 (IBM, Armonk, USA).

**Results:**

Thirty patients (73.3% female, 26.7% male) met the inclusion criteria, with an average age of 82.4 years. Analysis revealed a significant increase in kyphosis of the fractured vertebra in the thoracolumbar spine (*p* < 0.0001) following further sintering of osteoporotic VBF with increased lordosis caudal to the fracture (*p* < 0.0001). Total lumbar lordosis remained unchanged, alongside sacral slope measurements (*p* = 0.612 and *p* = 0.863, respectively).

**Conclusion:**

Progressive sintering of osteoporotic fractures in the thoracolumbar junction accentuates lordosis in underlying segments, potentially exacerbating degenerative changes and symptomatic manifestations. Thus, prioritizing interventions aimed at preventing progressive sintering and restoring sagittal balance is paramount in optimizing treatment outcomes for affected individuals.

## Introduction

Osteoporosis is the most common skeletal disease, affecting nearly 20% of the global population, with an incidence steadily increasing with advancing age [1]. Approximately one in five people will experience an osteoporotic vertebral body fracture (VBF) in their lifetime, causing significant morbidity and mortality in affected patients [2]. These fractures, whether managed conservatively or surgically, significantly influence the sagittal profile of the spine, with subsequent sintering potentially precipitating a progressive loss of sagittal balance [3, 4].

Alongside the aforementioned VBFs, a notable co-occurrence of fractures and other spinal degenerative disease manifests among elderly cohorts. Degenerative changes of the axial skeleton are often the underlying cause of back pain with facet joint arthritis affecting over half of individuals over the age of 65. Integral to spinal motion segments, facet joints and intervertebral discs undergo concurrent degeneration, culminating in pain and restricted motion within the affected segment and imposing undue stress upon adjacent regions [5, 6].

Loss of sagittal balance due to osteoporotic sintering fractures can exacerbate strain across motion segments throughout the spine. Therefore, previously asymptomatic degenerative changes frequently unmask clinical manifestations following vertebral fracture occurrence.

The pivotal role of lumbar sagittal alignment is well established in spinal surgery [7, 8], with an array of surgical interventions, including kyphoplasty and spondylodesis, aiming to rectify deviations from physiological alignment [9]. While extensive research has studied osteoporotic sintering fractures and degenerative spinal conditions, little attention has been directed towards comprehending the impact of progressive sintering of these fractures on the lumbar spine’s sagittal profile.

We hypothesize that progressive sintering of osteoporotic vertebral fractures in the thoracolumbar junction leads to a loss of the sagittal profile caused by an increased lumbar lordosis caudal to the fracture. This may cause previously asymptomatic degenerative changes in these segments to become symptomatic due to unphysiological loading. To address this, we conducted a retrospective analysis of radiographic lumbar and pelvic alignment in the context of progressive sintering of fractures in the thoracolumbar junction.

## Materials and methods

This investigation entailed a retrospective single center data analysis.

### Patient selection criteria

This study focused on patients who were admitted as inpatients or attended outpatient consultations for an osteoporotic fracture of the spine between 2017 and 2022. Patient data were queried by (International Statistical Classification of Diseases and Related Health Problems-10) ICD-10 codes, with subsequent sorting of the patient cohort conducted based on age in a descending manner.

### Inclusion and exclusion criteria

Inclusion criteria comprised patients diagnosed with a solitary osteoporotic vertebral body fracture localized within the thoracolumbar junction (TH11-L2), demonstrating evidence of further sintering during subsequent radiological assessments. Exclusion criteria were applied to patients exhibiting osteoporotic fractures outside the designated thoracolumbar region, lacking radiological follow-up data, displaying no discernible progressive sintering in follow-up examinations, manifesting new fractures during follow-up assessments, follow-up of less than 3 days, or having undergone operative interventions. Patients with incomplete datasets or lacking anteroposterior or lateral radiographs encompassing the entire thoracic and lumbar spine were also excluded.

### Data collection and radiographic analysis

Clinical and demographic parameters were meticulously recorded and subjected to analysis. Additionally, lateral-view radiographs of the patients were analyzed. The radiographic analyses were facilitated using the IDS 7-PACS^®^-System (Sectra, Linköping, Sweden). Measurements encompassed total lumbar lordosis (L1-5), lordosis caudal to the fracture, development of kyphosis of the fractured vertebra, and sacral slope. The vertebral angle was chosen instead of segmental kyphosis, which includes the disc, as this measurement specifically highlights how increasing kyphosis within the vertebral body itself impacts compensatory lordosis. This approach allows for future recommendations regarding fracture morphology to be developed.

**Fig. 1:**
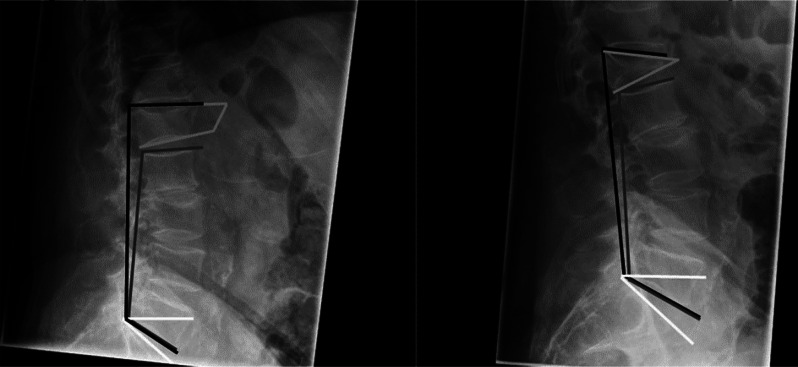
Exemplary measurements in lateral radiographs. Lateral radiographs demonstrate plotted measurements of the following spinal parameters: total lumbar lordosis (black), lordosis caudal to the fracture (dark grey), kyphosis of the fractured vertebra (light gray) and sacral slope (white)

Exemplary illustrations of the conducted measurements are shown in Fig. 1.

### Statistical analysis

Descriptive statistics are presented as mean values accompanied by the standard deviation (SD). Normal distribution of all continuous variables was assessed using Kolmogorov–Smirnov tests, with all variables demonstrating normal distribution. Subsequently, t-tests were employed for further analysis.

### Ethical considerations

This study was approved by the local ethics committee (Register number 2023–2357) and was conducted according to the revised Declaration of Helsinki.

## Results

### Patient population

Following application of stringent inclusion and exclusion criteria, a total of 30 patients were included in this study, median follow-up between radiographs was 62 days (IQR 300). Overall, 22 patients were female (73.3%), while 8 patients were male (26.7%). The average age of the patients enrolled in the study was 82.4 years, with a standard deviation of 6.5 years.

The distribution of fractured vertebral bodies among the included patients is outlined in Table 1.

**Table 1:** Distribution of the level of the fractured vertebral bodies from the included patients

Fractured Vertebral body	Number (absolute)	Number (relative)
TH 11	4	13.3%
TH 12	8	26.6%
L 1	11	36.7%
L 2	7	23.3%
Total	30	100%

### Kyphosis of the fractured vertebra and lumbar lordosis

The initial kyphosis in the region of the fractured vertebral body measured 10.4° (SD 1.0°) on primary radiographs. Subsequent follow-up assessments revealed an increase in this angle to 17.3° (SD 1.2°), representing a statistically significant increment of approximately 7° (*p* < 0.0001).

**Fig. 2:**
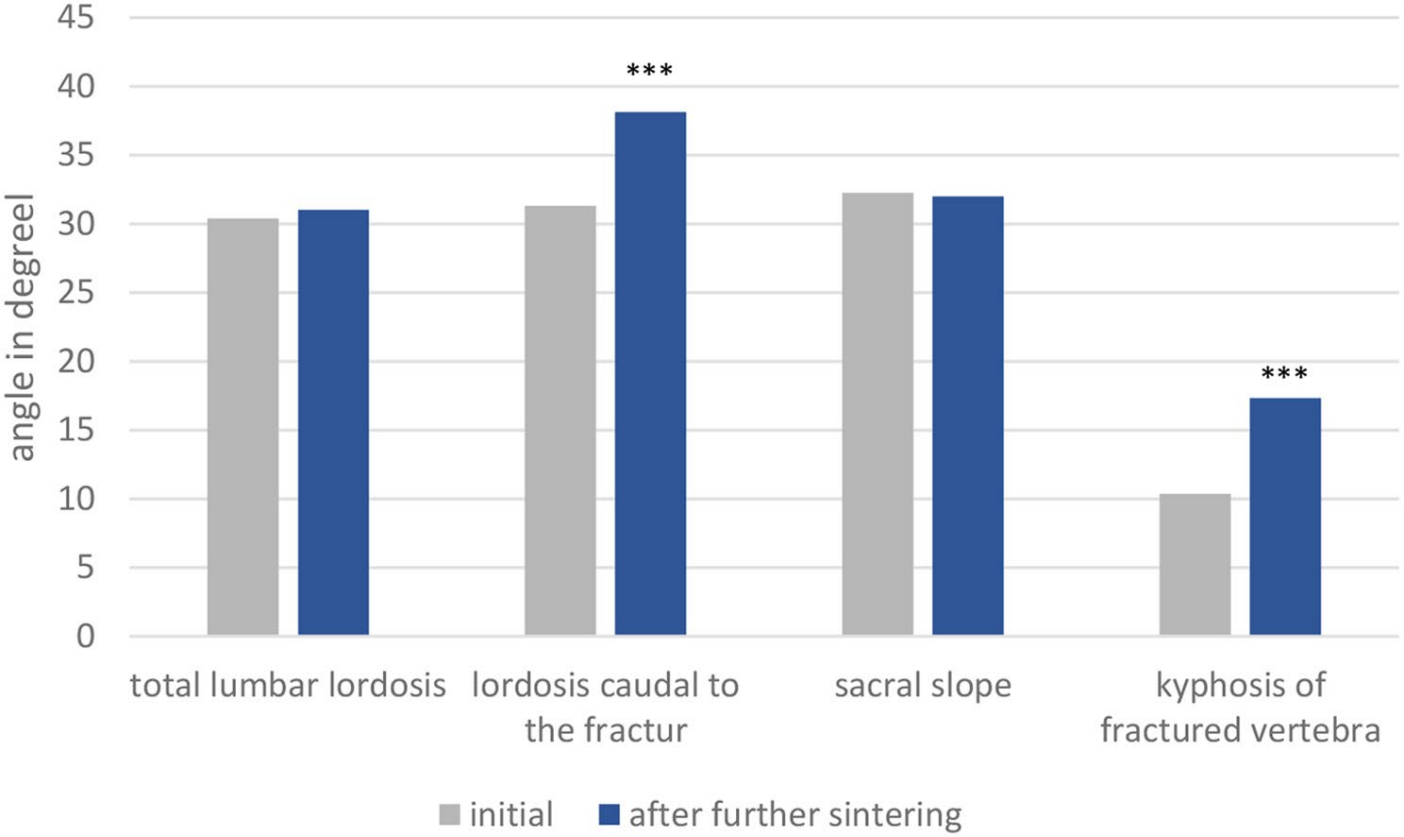
Comparison between total lumbar lordosis, lordosis caudal to the fracture, kyphosis of the fractured vertebra and sacral slope before and after further sintering

Furthermore, the lordosis in segments caudal to the fracture exhibited a noteworthy increase from 31.3° (SD 1.7°) in primary radiographs to 38.1° (SD 1.7°) in follow-up radiographs. This augmentation was also found to be statistically significant (*p* < 0.0001), as illustrated in Fig. 2.

Consequently, the total lumbar lordosis demonstrated minimal deviation, with no statistically significant difference observed between primary radiographs (30.4° ± 2.1°) and follow-up assessments (31.0° ± 2.0°) (*p* = 0.612).

Similarily, analysis of the sacral slope revealed no significant change between primary radiographs (32.3° ± 1.5°) and follow-up assessments (32.0° ±1.5°) (*p* = 0.863).

## Discussion

The progressive sintering of osteoporotic fractured vertebral bodies represents a common concern, particularly among elderly patients. The aim of this investigation was to analyze the effect of such progressive sintering of fractures in the thoracic lumbar junction on the sagittal lumbar profile. With an average patient age of 82.4 years and a notable female predominance, our study cohort aligns with the typical demographic affected by osteoporotic vertebral fractures [1, 2, 10–16].

Vertebral body fractures of the thoracolumbar junction are the most common spinal fractures [10, 11], owing to the region’s susceptibility to increased mechanical stress as a transition area from the more rigid thoracic spine to the mobile lumbar spine. In elderly individuals, age-related changes such as decreased intervertebral disc thickness and back muscle hypotrophy, coupled with progressive kyphosis, contribute to an anterior shift in the center of gravity. Consequently, this leads to increased compressive forces at the thoracic-lumbar junction, exacerbating vertebral collapse compared to other spinal levels [15, 16].

Our findings demonstrate a significant association between progressive sintering of vertebral fractures in this region and an ensuing increase in kyphosis of the fractured vertebra. This elevation in kyphosis imposes greater loads on vertebral endplates, potentially precipitating a cascade of vertebral fractures [16].

Clinically, it is often observed that initially asymptomatic degenerative changes of the spine manifest symptoms following osteoporotic fractures, likely attributed to alterations in sagittal profile induced by the fractures. Our study underscores this phenomenon, revealing a substantial increase in lordosis in underlying spinal segments following further sintering of osteoporotic vertebral fractures in the thoracolumbar junction (*p* < 0.0001).

While degenerative lumbar kyphosis typically correlates with extensive lumbar spine degeneration [13, 14], sparse evidence exists regarding the effect of progressive sintering of osteoporotic fractures on lumbar degeneration. Our findings elucidate a significant increase in kyphosis of the fractured vertebra, leading to hyperkyphosis in the thoracolumbar junction. As a result of this effect, we could demonstrate that further sintering of an existing osteoporotic fracture in the thoracolumbar junction leads to significant increase of lordosis in the underlying segments. It can be assumed that hyperlordosis in the underlying segments occurs as an attempt to compensate the hyperkyphosis in the thoracolumbal junction and to restore overall lumbar lordosis.

Patients with spondylarthritis have higher stress on their facet-joints. Consequently, there is a hypertrophy of these joints, which is one factor possibly leading to spinal stenosis. In these patients, hyperlordosis in the segments below an osteoporotic fracture causes further stress on the facet joint in these segments. Spinal canal stenosis in the lumbar segments can normally be compensated for by reduction of the lumbar lordosis. If now hyperlordosis occurs to compensate the loss of the sagittal balance which is caused by the osteoporotic fracture this might be an explanation for the phenomenon that degenerative changes, especially spinal stenosis, become symptomatically after the fracture even when they are settled a few segments caudal of the fracture.

Consequently, great emphasis should be placed on preventing progressive sintering and restoring vertebral body height, thereby preserving sagittal balance during treatment interventions. In this regard, attention should be paid to initiating an antiresorptive or osteoanabolic drug-based osteoporosis therapy tailored to the individual patient, with the greatest possible effect on spinal bone density [17, 18]. Additionally, in our opinion, the indication for the reduction of a sintering fracture in the thoracolumbar junction should be critically discussed, particularly with functionally demanding patients, in order to avoid progressive sintering and the potential consequences described above. It remains to be clarified at what degree of compression the indication for surgery should be considered. To address this, a risk score could be developed in prospective follow-up studies, integrating symptom-related and MRI data, to assess the likelihood of symptom progression following a sintering fracture. This score would help quantify the risk of symptomatic degenerative changes and enable precise guidance for patients.

Limitations of this study are primarily its retrospective design and the relatively small sample size. Due to the retrospective nature of the study, correlating clinical symptoms with radiographic findings was not feasible. Additionally, some patient specific parameters like BMI or medical history is not complete in our data. Thus, it could not be reported in a satisfying way.

Due to the quality of the available X-rays, pelvic parameters could not be analyzed in all cases. In the cases where analysis was possible, no significant changes in these parameters were observed. In future investigations on this topic, it may be beneficial to utilize full-spine X-rays to assess changes in thoracic kyphosis and pelvic parameters as well.

Moreover, there is a significant variation in the timing of when the follow-up radiograph was conducted. Additionally, the analysis focused solely on spinal alignment as observed in conventional radiographic images, without incorporating data on actual degenerative changes detectable in MRI scans. Therefore, future research should aim to validate the findings presented here through prospective studies utilizing MRI to provide a more comprehensive understanding.

## Conclusion

Progressive sintering of osteoporotic fractures in the thoracolumbar junction leads to significant increase of lordosis in the underlying segments. This phenomenon may exacerbate degeneration and symptomatology of pre-existing degenerative changes due to heightened strain and unphysiological loading in these segments. Thus, efforts to prevent progressive sintering and restore sagittal balance are crucial in treatment strategies for affected patients. Furthermore, consideration should be given to treating pre-existing spinal stenoses caudal to the fracture in order to achieve a good clinical result.

## Data Availability

No datasets were generated or analysed during the current study.

## Abbreviations

VBF: Vertebral body fracture
SD: Standard deviation
p: Level of significance
